# The Effect of Different Packaging Systems on the Shelf Life of Refrigerated Ground Beef

**DOI:** 10.3390/foods9040495

**Published:** 2020-04-14

**Authors:** Carlos A. Conte-Junior, Maria Lúcia G. Monteiro, Renata Patrícia, Eliane T. Mársico, Márcia M. Lopes, Thiago S. Alvares, Sérgio B. Mano

**Affiliations:** 1 Instituto de Química, Universidade Federal do Rio de Janeiro (UFRJ), Rio de Janeiro 21941-909, Brazil; marialuciaguerra@yahoo.com.br; 2 Núcleo de Análise de Alimentos (NAL-LADETEC), Universidade Federal do Rio de Janeiro (UFRJ), Rio de Janeiro 21941-598, Brazil; 3 Departamento de Tecnologia de Alimentos, Universidade Federal Fluminense (UFF), Niterói, Rio de Janeiro 24220-000, Brazil; ceicao_5@yahoo.com.br (R.P.); elimarsico@gmail.com (E.T.M.); marcia.vet@globo.com (M.M.L.); mtasbm@vm.uff.br (S.B.M.); 4 Instituto Nacional de Controle de Qualidade em Saúde, Fundação Oswaldo Cruz (FIOCRUZ), Rio de Janeiro 21040-900, Brazil; 5 Instituto de Nutrição, Universidade Federal do Rio de Janeiro (UFRJ), Rio de Janeiro 27979-000, Brazil; alvares@macae.ufrj.br

**Keywords:** minced meat, modified atmosphere packaging, MAP, vacuum packaging, physicochemical quality indicators, predictive microbiology

## Abstract

The aim of this study was to investigate the effects of different packaging systems on the shelf life of refrigerated ground beef. The ground beef samples were packaged as follows: AA (100% ambient air), 90O_2_:10CO_2_ (90% O_2_ and 10% CO_2_), 80O_2_:20CO_2_ (80% O_2_ and 20% CO_2_), 70O_2_:30CO_2_ (70% O_2_ and 30% CO_2_), 60O_2_:40CO_2_ (60% O_2_ and 40% CO_2_), 50O_2_:50CO_2_ (50% O_2_ and 50% CO_2_), 100O_2_ (100% O_2_), and VP (vacuum packaging). All treatments were analyzed daily for O_2_ and CO_2_ levels, pH, filtration time, total volatile basic nitrogen (TVB-N), aerobic mesophilic heterotrophic bacteria (AMHB), and aerobic psychrotrophic heterotrophic bacteria (APHB) over 20 days at 2 °C. All MAP systems had a decrease of O_2_ and an increase of CO_2_ levels during storage period (*p* < 0.05). Overall, the MAP systems were similarly able to decrease the pH and retard the increase of TVB-N and filtration time over the storage period (*p* > 0.05). Moreover, the MAP systems increased the lag phase and/or the generation time of both AMHB and APHB, extending the shelf life by 3 (90O_2_:10CO_2_), 4 (70O_2_:30CO_2_ and 100O_2_), and 5 days (80O_2_:20CO_2_, 60O_2_:40CO_2_, 50O_2_:50CO_2,_ and VP). All MAP systems were equally effective in retarding physicochemical degradation; however, 80O_2_:20CO_2_, 60O_2_:40CO_2_, 50O_2_:50CO_2,_ and VP were the most effective in impairing bacterial growth and extending the shelf life of ground beef stored under refrigeration.

## 1. Introduction

Brazil is one of the main world beef producers; therefore, it has been investing in alternative technological strategies to produce high-quality beef products and meet consumers’ requirements [1]. Beef products, mainly ground beef, are considered ideal substrates for spoilage and pathogenic bacteria growth, resulting in a rapid loss of quality and limited shelf life [2]. This fact, together with an increased demand for the use of nonthermal processing technologies as an alternative for preserving the meat’s original quality, makes it necessary to find new technological approaches for ground beef preservation [3,4]. Brazilian beef cuts are commonly displayed in vacuum packages in retail displays [1]. However, the color of vacuum-packed meat (dark purplish red) is unpleasant to consumers. In this way, modified atmosphere packaging (MAP) is an interesting alternative for the meat industry due to its capacity to extend shelf life and maintain the original quality parameters of fresh beef cuts until consumption [5,6]. Nonetheless, in Brazil, there is still limited information related to the application and effectiveness of MAP for some beef products. The main gases used for the MAP of fresh beef are carbon dioxide (CO_2_), oxygen (O_2_), and nitrogen (N_2_). MAP with high levels of O_2_ (70–80%) and level of CO_2_ between 20–30% are widely used due to their effectiveness in reducing microbial growth and maintaining the red meat color desired by global consumers [1]. However, MAP with high levels of O_2_ may accelerate lipid oxidation, leading to the formation of undesirable off-flavor compounds [7], as well as protein oxidation, causing adverse effects on the tenderness and juiciness of beef products [8,9].

Some authors have evaluated the influence of different concentrations of CO_2_ and O_2_ on the quality parameters of fresh beef, including minced meat, which is more perishable due to its grinding process [10,11,12,13,14]. It is worth highlighting that every meat cut has its own intrinsic chemical characteristics and normal microbiota, which are explicitly influenced when gas mixtures are used. Furthermore, due to microbial metabolism and the partial pressure and solubility of gases under different CO_2_ and O_2_ ratios [15,16], each product has an optimal gas composition, which is a key factor and major challenge for the successful application of MAP. However, at the present moment, there are no studies evaluating the effect of MAP over a wide range of gas compositions in ground beef from the *Serratus ventralis thoracis* muscle, which is used commonly to make ground beef in Brazil.

In this context, the aim of this study was to investigate the effects of different packaging systems, such as air ambient, vacuum packaging, and six different types of O_2_/CO_2_ blend-MAPs, on the shelf life of fresh ground beef kept under refrigerated conditions (2 °C) for 20 days.

## 2. Materials and Methods

### 2.1. Beef Sampling

Nellore (*Bos indicus*) bulls were slaughtered in a local federal inspected abattoir (São João de Meriti, Rio de Janeiro, Brazil). The carcasses were maintained cool at 0 °C. *Serratus ventralis thoracis* (SVT) muscles were sampled 24 h *post-mortem* from the left halves of the carcasses, deboned manually at 12 °C, and the excess fat, aponeuroses, ligaments, tendons, and lymph nodes were removed. The SVT muscles were then cut into steaks, vacuum-packed, and conveyed to the grinding section in an air-conditioned environment at 5 °C.

### 2.2. Preparation of Ground Beef

Steaks cut were ground using a large-scale stainless steel meat grinder (AFMG300, Hess Meat Machines, St. Louis, MO, USA) equipped with a 13 mm mesh filter plate and a working capacity up to 9000 kg/h. The obtained ground beef was then conveyed to the mixer (DMX300, Hess Meat Machines, USA) equipped with one paddle. During mixing, dry ice was injected into the ground beef through jets mounted at the bottom of the mixer. The temperature of the ground beef at the mixer outlet was reduced to around −0.7 °C.

For further mincing, the ground beef was ground through a second stainless steel meat grinder (AFMG50, Hess Meat Machines, USA) equipped with a 3.2 mm mesh filter plate with a working capacity up to 1800 kg/h. The resulting ground beef at the grinder output was divided into equal portions of 0.8 kg using an electronic balance (AW6200GP, Hess Meat Machines, USA) and conveyed for packaging.

### 2.3. Packaging and Storage of Ground Beef

Ground beef samples were packed into heat-shrink Cryovac^®^-BB4L bags, composed principally of polyolefine and polyvinylidene chloride layers, with a thickness of 102 μm and gas permeability (at 25 °C) of 62.5 cm^3^/h/m/MPa for CO_2_, 14.6 cm^3^/h/m/MPa for O_2_, and 0.6 cm^3^/h/m/MPa for N_2_, as provided by the manufacturer. A heat-shrink pack sealer (model Sipromac Vac 300, Hess Meat Machines, USA) equipped with a vacuum chamber was used for the packaging and injection of gases that were mixed and certified by a WITT-Gasetechnik device (WITT-Gasetechnik GmbH and Co KG, Witten, Germany). The gases O_2_ and CO_2_ were supplied from Linde AGA (Lohne, Germany). The ground beef samples were packaged as follows: AA (100% ambient air), 90O_2_:10CO_2_ (90% O_2_ and 10% CO_2_), 80O_2_:20CO_2_ (80% O_2_ and 20% CO_2_), 70O_2_:30CO_2_ (70% O_2_ and 30% CO_2_), 60O_2_:40CO_2_ (60% O_2_ and 40% CO_2_), 50O_2_:50CO_2_ (50% O_2_ and 50% CO_2_), 100O_2_ (100% O_2_), and VP (vacuum packaging). The packed ground beef samples were then held at 2 °C in a conventional refrigerator coupled to an internal digital thermometer (TH 439, Equitherm, Rio de Janeiro, Brazil) with a scale ranging from −10 °C to 50 °C to monitor the temperature of the samples during all storage periods (20 days). The samples were then analyzed for pH, filtration time, and TVB-N, AMHB, and APHB counts. The criterion for determining the days of analyses was based on obtaining the stationary phase of both bacterial groups (AMHB and APHB) for each treatment according to the predictive primary model designed by Baranyi and Roberts [17] through the DMFit program version 2.0 (Institute of Food Research, Norwich, UK). This occurred on day 9 for AA and on day 20 for all other treatments. Therefore, AA was evaluated daily from day 0 to 9, while 90O_2_:10CO_2_, 80O_2_:20CO_2_, 70O_2_:30CO_2_, 60O_2_:40CO_2_, 50O_2_:50CO_2_, 100O_2,_ and VP were evaluated daily from day 0 to 20. In the experiment, 48 h *post-mortem* is day 0. In addition, it is worth noting that ground beef is usually manufactured from beef cuts of a lower quality, such as *Serratus ventralis thoracis*. Nevertheless, there are no studies investigating the effects of different packaging systems, including MAP, on ground beef from this beef muscle.

### 2.4. Gas Analysis

Gas analysis of the internal atmosphere was carried out every storage day using a digital O_2_/CO_2_ headspace gas analyzer (OXYBABY^®^, WITT-Gasetechnik GmbH and Co KG, Witten, Germany) by withdrawing a 10 mL gas sample through a septum glued onto the surface of the pack using the analyzer’s needle [13].

### 2.5. Physicochemical Analyses

The pH values were measured through a digital pH meter (K39-1014B, Kasvi, Paraná, Brazil) by direct insertion of the electrode into the sample [18].

Total volatile basic nitrogen (TVB-N) was determined in 10 g of ground beef using the Conway micro-diffusion method according to the protocol established by the Association of Official Analytical Chemists [19].

The alteration of the freshness and integrity of the myofibrils was assessed using a filtration test. Ten grams of ground beef sample homogenized into 100 mL of distilled water were filtered using Whatman paper No. 40 [20,21], and the results were obtained by timing the filtration time.

### 2.6. Bacteriological Analyses

The aerobic mesophilic heterotrophic bacteria (AMHB) and aerobic psychrotrophic heterotrophic bacteria (APHB) in the meat sample were analyzed using the standard methods of the American Public Health Association [22]. Samples of ground beef (25 g) in the stomacher bags were aseptically added to 225 mL of sterile buffered peptone water solution (0.1% *w*/*v*) and homogenized for 2 min at 25 °C. After successive decimal dilutions, a suitable dilution 100 μL in volume was applied on the surfaces of the agar plates. AMHB and APHB were determined on a plate count agar (PCA; Difco Laboratories, Detroit, MI, USA), incubated at 35 °C for 48 h, and then at 7 °C for ten days. Plates enclosing from 25 to 250 colonies were chosen, and the average number of cfu/g was calculated. Bacterial colonies were considered and expressed as Log cfu (colony forming units) per gram of ground beef.

### 2.7. Statistical Analyses

The number of total samples analyzed was 298, and all analyses were performed in duplicate [1 (AA treatment) × 9 (days of storage) × 2 (duplicate) + 7 (90O_2_:10CO_2_, 80O_2_:20CO_2_, 70O_2_:30CO_2_, 60O_2_:40CO_2_, 50O_2_:50CO_2_, 100O_2_ and VP treatments) × 20 (days of storage) × 2 (duplicate)]. The relationship between each pH, TVB-N, filtration time, and days of storage was separately analyzed for each treatment through a linear regression analysis. A one-way ANOVA and Tukey’s post hoc test was used to identify differences in the total amount of each physicochemical parameter produced during the storage period between the treatments. The bacterial growth curves were fit according to a predictive primary model using the statistical program DMFit 2.0 (Institute of Food Research, Norwich, UK) designed by Baranyi and Roberts [17]. This program was also used to obtain the bacterial growth parameters (lag phase—Lag, generation time—GT, and number of colonies in the stationary phase—NC) of each treatment, which were further evaluated by a one-way ANOVA with a Tukey post-hoc test. All analyses were performed using the XLSTAT software, version 2012.6.08 (Addinsoft, New York, NY, USA), at a 0.05 level of confidence (*p* < 0.05).

## 3. Results and Discussion

### 3.1. Headspace Gas Levels in the MAP Samples

**Figure 1:**
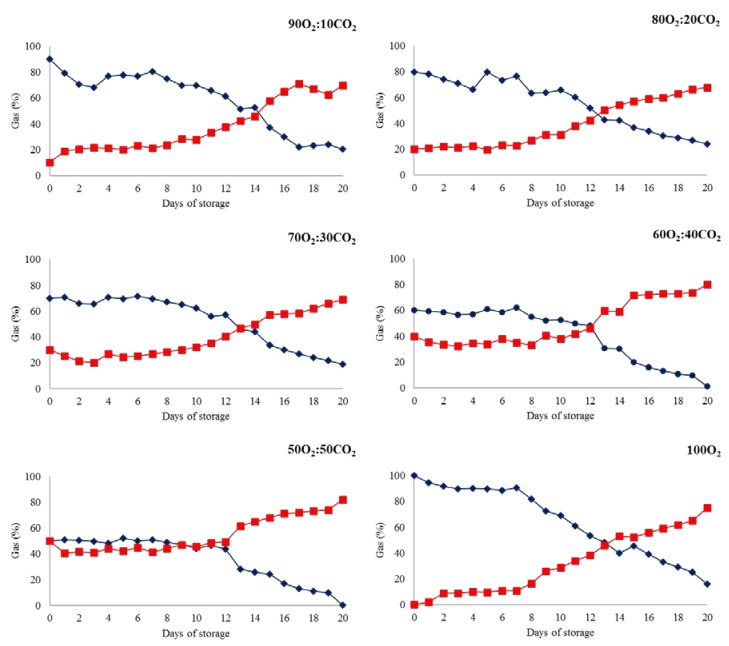
Effect of storage time on the headspace O_2_ (blue color) and CO_2_ levels (red color) of ground beef treated with different packaging systems stored at 2 °C for 20 days. 90O_2_:10CO_2_, 80O_2_:20CO_2_, 70O_2_:30CO_2_, 60O_2_:40CO_2_, 50O_2_:50CO_2_, and 100O_2_ (modified atmosphere packaging with 90%:10%, 80%:20%, 70%:30%, 60%:40%, 50%:50%, and 100%:0% oxygen and dioxide carbon ratios, respectively).

A significant reduction of O_2_ and an increase in CO_2_ levels (*p* < 0.05) were observed for all the MAP types (Figure 1). This behavior could be related to the dominant bacteria in the refrigerated meat packed with MAP [23]. While *Pseudomonas* sp. utilize available oxygen in the headspace, facultative anaerobic lactic acid bacteria, such as *Brochothrix thermosphacta*, and lactic acid bacteria (LAB) produce carbon dioxide as a metabolic product, causing a reduction of O_2_ and the emission of CO_2_ into packages during the storage period [13,23,24]. Similarly, the emission of CO_2_ and consumption of O_2_ were also observed in previous studies [13,25,26,27].

Regarding the rate of O_2_ reduction, the plots of O_2_ and CO_2_ levels crossed over approximately at the same storage time (13–14 days), despite their different initial O_2_ and CO_2_ levels. The intersections of the O_2_ and CO_2_ plots had approximately the same O_2_ and CO_2_ levels, ranging from 46% to 50% (*v*/*v*). The barrier property of the packaging material is likely the reason for this behavior. Indeed, the oxygen permeability of the Cryovac^®^-BB4L bag depends on the differential partial pressure of O_2_ between the internal and external sides of the packaging material. After a certain time, gas composition in the package of the ground beef reaches a definite balance between the respiration rate and permeability of the packaging material. In this state of equilibrium, the total amounts of CO_2_ emitted and O_2_ consumed by respiration are the same as those permeated through the packaging material exchange [28]. The respiration of ground beef, storage environmental factors (i.e., temperature and relative humidity), and the permeability of the packing materials determine the gas composition at this equilibrium state after a storage time of 13–14 days [13,28].

### 3.2. Physicochemical Parameters

During the entire period of storage, AA showed an increase, while the other treatments demonstrated a decrease in pH values (*p* < 0.05; Table 1). AA had the highest pH value (*p* < 0.05), and no difference (*p* > 0.05) was observed among the remaining treatments until the 9th day of storage. Amongst packaging system treatments from the 10th to 20th day of storage, 100O_2_ had a higher pH value than VP, 60O_2_:40CO_2,_ and 50O_2_:50CO_2_ (*p* < 0.05), and VP showed the lowest value for this parameter (*p* < 0.05), except when compared to 50O_2_:50CO_2_ (*p* > 0.05; Table 1).

**Figure 2:**
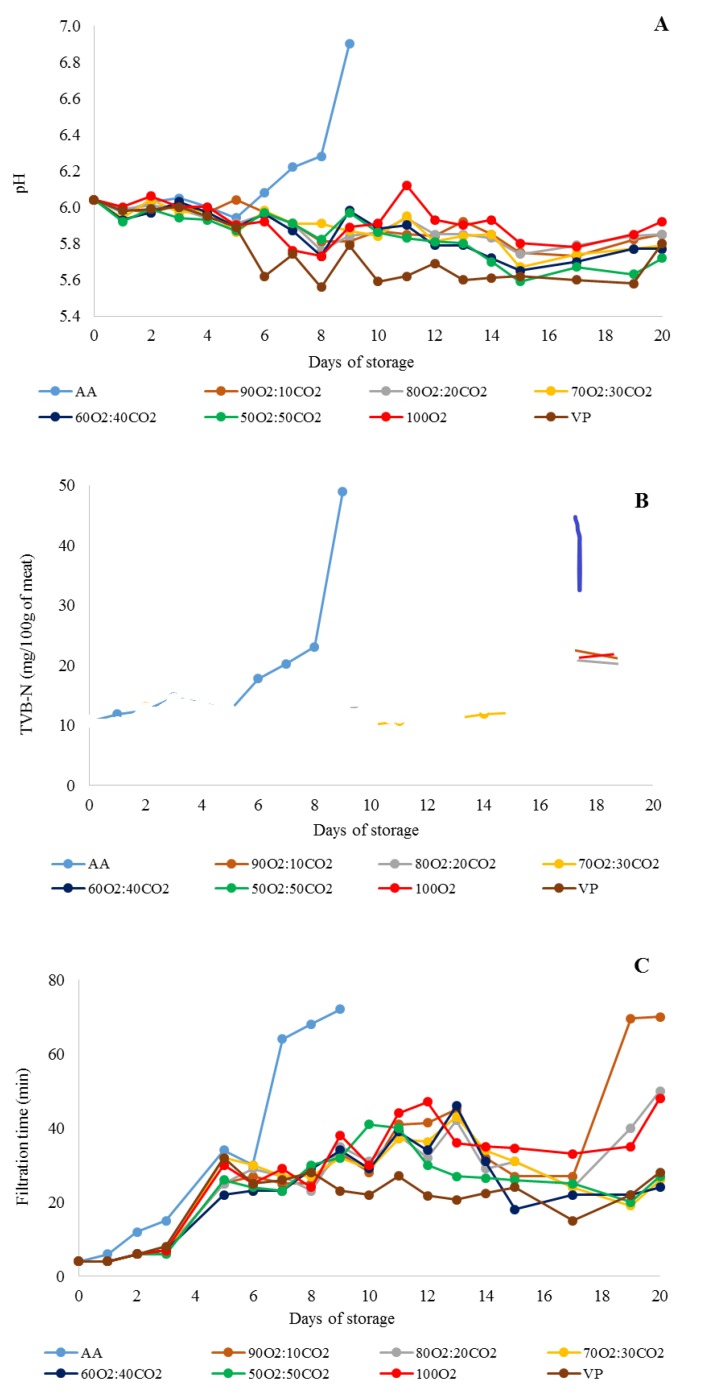
Results of pH: (**A**) total volatile basic nitrogen (mg of TVB-N/100 g of meat); (**B**) and filtration time (min); (**C**) of the ground beef treated with different packaging systems stored at 2 °C for 20 days. AA (ambient air); 90O_2_:10CO_2_, 80O_2_:20CO_2_, 70O_2_:30CO_2_, 60O_2_:40CO_2_, 50O_2_:50CO_2_, and 100O_2_ (modified atmosphere packaging with 90%:10%, 80%:20%, 70%:30%, 60%:40%, 50%:50%, and 100%:0% of oxygen and dioxide carbon ratios, respectively); VP (vacuum packaging).

Brazilian regulations declare 6.4 as the maximum tolerated pH of meat destined for human consumption [29]. The initial pH value of ground beef was 6.1 (Figure 2A). AA exceeded this limit on day 9 when it reached a pH value of 6.8. On the other hand, all other treatments had a pH ranging from about 6.10 to 5.80 and, therefore, did not achieve a pH value of 6.4 throughout refrigerated storage.

The increase in the pH value for AA might be attributed to the accumulation of basic compounds derived from the growth of *Pseudomonas* spp. and associated sub-species [30]. The decrease of the pH value in 10–50% CO_2_ MAP treatments may be attributed to the formation of carbonic acid by the dissolution of CO_2_ in water [31]. In vacuum packaging, a decrease in pH values may be due to low O_2_ levels favoring the growth of acid lactic bacteria, which are facultative anaerobic bacteria [32,33]. Similar findings were previously reported in the literature for meat [34,35].

The TVB-N parameter is utilized as a food freshness indicator, since volatile nitrogen-based compounds are the product of the degradation of protein and non-protein nitrogen compounds, such as trimethylamine (TMA) and ammonia, which are mainly associated with the growth of spoilage bacteria [36]. Although the TVB-N values increased in all treatments during the entire storage period, this increase occurred more rapidly in AA (Table 1). AA showed the highest TVB-N values (*p* < 0.05) until the 9th day of storage, and the remaining treatments were similar for this parameter during all storage periods (*p* > 0.05; Table 1).

The initial TVB-N value was 10.70 mg of TVB-N/100 g, which is acceptable according to the standards recommended by Brazilian regulations. However, AA exhibited 49.00 mg of TVB-N/100 g on the 9th day of storage, which is higher than the standard limits (≤30 mg of TVB-N/100 g) [28]. All other treatments did not reach this limit throughout the refrigerated storage period, producing values between 18.55 and 23.98 mg of TVB-N/100 g on the last day of storage (Figure 2B).

Our results revealed that both O_2_/CO_2_ enriched atmospheres and vacuum packaging reduced the growth of spoilage bacteria, probably due to the antibacterial activity of CO_2_ and the high levels or absence of O_2_ [30,33,37]. In agreement with our findings, some authors have already reported that MAP with different O_2_ and CO_2_ ratios and vacuum packaging can delay the formation of TVB-N by decreasing the growth rate of spoilage bacteria in refrigerated meat [36,38]. Furthermore, the TVB-N analysis proved to be a useful indicator for monitoring the freshness of ground beef, although further analyses should be performed in order to establish reference values for ground beef using this parameter.

The filtration time increased under all treatments over the storage period. However, the filtration time was much more pronounced in AA (*p* < 0.05; Table 1). Until the 9th day of storage, AA showed the highest filtration time (*p* < 0.05), and the remaining treatments were similar for this parameter (*p* > 0.05). From the 10th to 20th day of storage, VP demonstrated a lower filtration time (*p* < 0.05) than that of 90O_2_:10CO_2_ and 100O_2_, which did not differ from each other (*p* > 0.05). Likewise, the 20–50% CO_2_ MAP treatments had similar filtration times to 90O_2_:10CO_2,_ 100O_2,_ and VP (*p* > 0.05).

According to Brazilian regulations [20], a filtration time of 5 min indicates fresh meat suitable for consumption, 6–10 min indicates a meat of medium quality, and ≥10 min means an altered meat that is not suitable for consumption. AA exceeded 10 min of filtration time on day 3 of refrigerated storage (15 min), while all other treatments exceeded this limit on day 5 (22–32 min; Figure 2C). On the 9th day of storage, AA showed 72 min of filtration time, and the remaining treatments had filtration times ranging from 23 to 38 (Figure 2C).

The filtration time provides an indirect indication of the water retention capacity of the meat sample, which is also related to the structural integrity of the myofibrils of the ground beef [39]. The proteolytic action of endogenous proteases is responsible for the disorganization of proteins and connective tissue in ground beef. Therefore, in general, a high filtration time indicates a high water holding capacity, which is an indication of protein denaturation and supports the proteolysis of myofibrillar proteins. Changes in the intracellular architecture of fibrils can be induced by autolytic action and can influence the ability of proteins to retain water [40,41]. Degradation of these proteins allows water to remain in the cell for a more extended period. This tenderization is an enzymatic alteration; thus, physiochemical conditions may modify the proteolytic activity of endogenous enzymes [42]. These results demonstrate that the effects of different atmosphere systems (several O_2_ and CO_2_ ratios and vacuum packaging) might be responsible for the modulation of pH (Table 1), thereby slowing down the proteolysis and disorganization of the myofibrillar structure, confirmed by the filtration test results [43].

### 3.3. Bacterial Growth

**Figure 3:**
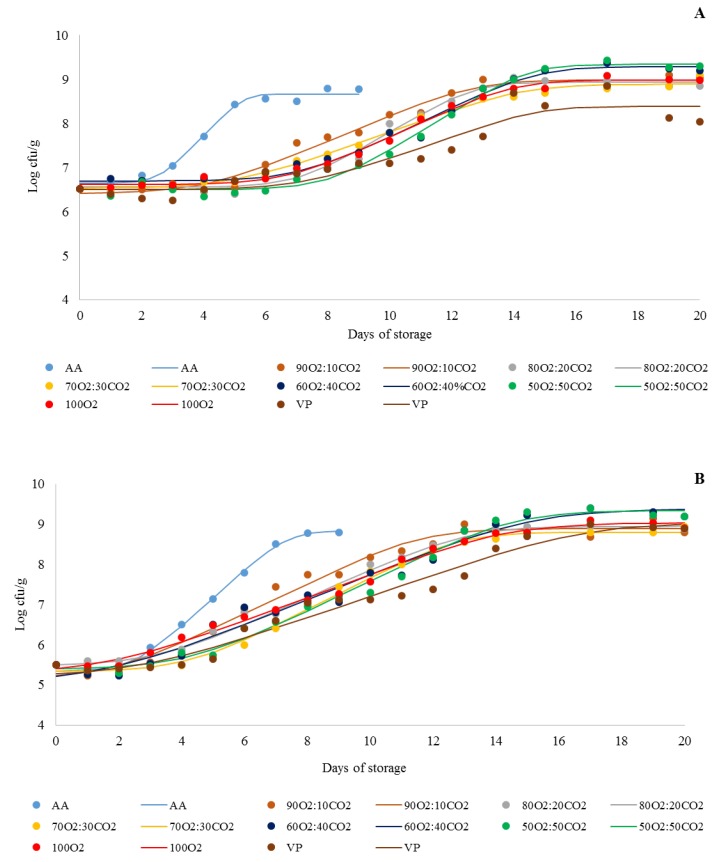
Growth curve of aerobic mesophilic heterotrophic bacteria (**A**) and aerobic psychrotrophic heterotrophic bacteria (**B**) of ground beef treated with different packaging systems stored at 2 °C for 20 days. AA (ambient air); 90O_2_:10CO_2_, 80O_2_:20CO_2_, 70O_2_:30CO_2_, 60O_2_:40CO_2_, 50O_2_:50CO_2_, and 100O_2_ (modified atmosphere packaging with 90%:10%, 80%:20%, 70%:30%, 60%:40%, 50%:50%, and 100%:0% oxygen and dioxide carbon ratios, respectively); VP (vacuum packaging). Log cfu/g—Log colony forming units per gram. Filled circles indicate the real average values (*n* = 2), and lines represent the values fitted by the predictive primary model designed by Baranyi and Roberts [17].

The bacterial growth curves and growth parameters (Lag, GT, and NC) are shown in Figure 3 and Table 2, respectively. The initial count of AMHB was 6.5 Log cfu/g of ground beef (Figure 3A). This initially high bacterial count might be due to the inadequate handling of meat samples during slaughtering, cutting, and processing [38]. Moreover, ground beef has a high exposed surface, which results in higher microbial contamination than the surface of whole meat [44].

The AMHB (Figure 3A) and APHB (Figure 3B) counts of the ground beef increased with storage time in all treatments. To determine the shelf life of stored ground beef, we adopted a maximum value of 7.0 Log cfu/g for mesophilic bacterial counts (established by the ICMSF [45]) as a microbiological standard for meat products safe for consumption.

The counts of AMHB in AA grew much faster than those of the other groups, reaching the standard shelf life threshold (7.0 Log cfu/g) [45] on the 3rd day of refrigerated storage (Figure 3A; Table 2). In relation to all other treatments, the AMHB count of 7.0 Log cfu/g was achieved on days 6 (90O_2_:10CO_2_), 7 (70O_2_:30CO_2_ and 100O_2_), and 8 (80O_2_:20CO_2_, 60O_2_:40CO_2_, 50O_2_:50CO_2_ and VP). These results may be explained by the effect of the different packaging systems on the bacterial growth parameters. According to Baranyi and Roberts [17], the lag phase is calculated as the time necessary during which bacterial cells modify themselves in order to initiate exponential growth. The generation time is calculated during the exponential phase of growth as the time need for bacterial count to double through growth rate (μmax) by the formula GT = log (2)/μmax. The stationary phase is calculated as the highest final bacterial count during storage.

AA had the lowest lag phase and generation time (GT; *p* < 0.05), resulting in a shorter shelf life. Although 90O_2_:10CO_2_ and 70O_2_:30CO_2_ showed a higher GT, they demonstrated a lower lag phase compared to 80O_2_:20CO_2_, 60O_2_:40CO_2,_ and 50O_2_:50CO_2_ (*p* < 0.05), indicating that the mesophilic bacteria group grew more slowly but adapted more quickly in 90O_2_:10CO_2_ and 70O_2_:30CO_2_. 100O_2_ and 60O_2_:40CO_2_ had similar GT values (*p* > 0.05). However, 60O_2_:40CO_2_ demonstrated a higher lag phase than 100O_2_ (*p* < 0.05). In addition, although VP exhibited a similar GT to 70O_2_:30CO_2_, its lag phase was as long as 60O_2_:40CO_2_ (*p* > 0.05).

Likewise, 80O_2_:20CO_2_, 60O_2_:40CO_2_, 50O_2_:50CO_2_, 100O_2,_ and VP also had higher lag phases or GTs for their APHB counts. 80O_2_:20CO_2_ and 50O_2_:50CO_2_ showed higher lag phases than 90O_2_:10CO_2_ (*p* < 0.05). VP had the highest GT, followed by 100O_2_, 60O_2_:40CO_2_, 80O_2_:20CO_2,_ and 50O_2_:50CO_2_ (*p* < 0.05). Although 50O_2_:50CO_2_ showed a similar GT to 90O_2_:10CO_2,_ 50O_2_:50CO_2_ had the highest lag phase among all treatments (*p* < 0.05). 80O_2_:20CO_2_ and VP had a higher lag phase than 90O_2_:10CO_2_ and 100O_2_ (*p* < 0.05). Furthermore, 70O_2_:30CO_2_ demonstrated a higher lag phase than all other treatments, except for 50O_2_:50CO_2_. However, 70O_2_:30CO_2_ had the lowest GT among the MAP treatments (*p* < 0.05). Furthermore, the highest viable cells in the stationary phase (NC) were found in 60O_2_:40CO_2_ and 50O_2_:50CO_2_ for both the AMHB and APHB groups (*p* < 0.05). This fact may be attributed to the sublethal injury to bacterial cells induced by different O_2_ and CO_2_ ratios, leading to injured cells initially growing slower than intact cells, followed by the rapid growth of recovered cells, mainly in a medium without natural competition [46,47].

According to our results, the O_2_/CO_2_-MAP is proven to have an antibacterial effect [23,48,49]. The high solubility of CO_2_ in water and fat leads to the formation of carbonic acid and a reduction in meat pH, resulting in an unfavorable acidic environment for bacterial growth [31,50]. The use of O_2_ is necessary in beef to maintain its attractive red color [1], and, despite the direct bacteriostatic effect from CO_2_, O_2_ influences the growth of different bacterial groups depending on its levels [34,35]. Therefore, different CO_2_ and O_2_ ratios result in changes in microbial metabolism and subsequent differences in the partial pressure of gases, which is directly related to gas solubility and is a key factor in the antimicrobial effectiveness of MAP systems. Furthermore, the effect of different CO_2_ and O_2_ ratios also depends on the intrinsic chemical characteristics of the meat cut, such as its proximate composition [15,16]. According to Hunt et al. [51], *Serratus ventralis* muscles contain 9.84% lipids, 20.52% proteins, and 67.61% moisture. In this way, the largest challenge to the successful application of MAP is to find the optimal gas composition for each food product since MAP systems with higher CO_2_ levels are not necessarily the most effective ones and are mainly used for food with a high surface area, such as ground beef. Therefore, studies focusing in the knowledge about the effects of MAP with different gas ratios in beef muscle, which are widely used to make ground beef but not studied yet, as the present study, contribute strongly to industrial MAP application. In partial agreement with our findings, Yang et al. [52] reported similar effects in two MAP systems (80% O_2_ + 20% CO_2_ and 50% O_2_ + 30% CO_2_ + 20% N_2_) against bacterial growth in beef steaks during 12 days of refrigerated storage.

VP demonstrated results comparable to some O_2_/CO_2_-MAP systems, which may be explained by the removal of oxygen, which inhibits the growth of obligate aerobic bacteria [34,35]. These results corroborate those of previous studies that demonstrated an equal or better bacterial quality of meat under vacuum conditions compared to MAP containing different levels of O_2_ and CO_2_ [52,53]. The growth of aerobic bacteria in VP samples may be attributed to the growth of acid lactic bacteria, which grows in the absence or presence of O_2_, mainly in environments without natural competition [32,33]. Moreover, vacuum packaging retains approximately 5% residual oxygen due to the inability to completely remove O_2_ and the penetration of O_2_ through the packaging during the storage period [54,55]. Nevertheless, vacuum packaging cause changes to the meat color (creating a purple color), which is undesirable to consumers [56]. On the other hand, O_2_ may induce lipid oxidation, one of the main non-microbiological factors leading to meat deterioration during refrigerated storage. This phenomenon causes a loss of nutrients, off-flavor, discoloration, limited shelf life, and the formation of compounds harmful to human health [7,57]. Therefore, further studies are necessary to evaluate the oxidative stability of ground beef submitted to MAP 50–80% O_2_ levels and VP throughout refrigerated storage.

The results found for 100O_2_ may be attributed to the high concentrations of O_2_ due to the required O_2_ levels (around 21%) for optimal bacterial growth [58]. The toxicity of high O_2_ to aerobic bacteria may be associated with the formation of superoxide radicals (O_2_^−^) [59].

## 4. Conclusions

All MAP packaging systems equally delay the loss of physicochemical quality during refrigerated storage of ground beef. However, the predictive microbiological parameters revealed that the most effective MAP systems were 80O_2_:20CO_2_, 60O_2_:40CO_2_, 50O_2_:50CO_2,_ and VP, which extended the shelf life of the ground beef by five days, while 90O_2_:10CO_2_, 70O_2_:30CO_2,_ and 100O_2_ extended the shelf life by 3, 4, and 4 days, respectively. VP was as effective as 80O_2_:20CO_2_, 60O_2_:40CO_2,_ and 50O_2_:50CO_2_. However, VP is known to change the color of fresh beef, which is an essential attribute for consumer acceptance of red meat products. Therefore, 80O_2_:20CO_2_, 60O_2_:40CO_2,_ and 50O_2_:50CO_2_ offer a simple and effective method to preserve the physicochemical quality and enhance the shelf life of ground beef stored at 2 °C for 20 days. Further studies should be performed to evaluate the oxidative potential of MAP with 50–80% O_2_ levels for minced meat.

## Figures and Tables

**Table 1:** Physicochemical parameters of the ground beef treated with different packaging systems stored at 2 °C for 20 days.

Parameters	Treatments ^€^	∆_0–20_^£^		Linear Regression Coefficients			
		∆_0–9_	∆_10–20_	y-Intercept	Slope	*p*-Value	*r*-Squared
pH	AA	6.15 ± 0.28 ^a^	NA	5.85 ± 0.13	0.07 ± 0.02	0.0230	0.496
	90O_2_:10CO_2_	5.95 ± 0.08 ^b^	5.83 ± 0.06 ^a,b^	6.01 ± 0.02	−0.01 ± 0.00	<0.0001	0.644
	80O_2_:20CO_2_	5.94 ± 0.08 ^b^	5.84 ± 0.05 ^a,b^	5.99 ± 0.02	−0.01 ± 0.00	0.0000	0.596
	70O_2_:30CO_2_	5.95 ± 0.06 ^b^	5.81 ± 0.08 ^a,b^	6.01 ± 0.02	−0.01 ± 0.00	<0.0001	0.715
	60O_2_:40CO_2_	5.94 ± 0.09 ^b^	5.77 ± 0.08 ^b^	6.01 ± 0.03	−0.02 ± 0.00	<0.0001	0.650
	50O_2_:50CO_2_	5.94 ± 0.06 ^b^	5.73 ± 0.10 ^b,c^	6.01 ± 0.03	−0.02 ± 0.00	<0.0001	0.779
	100O_2_	5.93 ± 0.11 ^b^	5.90 ± 0.10 ^a^	5.99 ± 0.04	−0.01 ± 0.00	0.0420	0.204
	VP	5.86 ± 0.17 ^b^	5.63 ± 0.07 ^c^	5.94 ± 0.05	−0.02 ± 0.00	0.0000	0.531
TVB-N ^¥^	AA	18.64 ± 11.39 ^a^	NA	5.82 ± 4.63	2.85 ± 0.87	0.0110	0.574
	90O_2_:10CO_2_	11.45 ± 1.98 ^b^	16.26 ± 4.58 ^a^	8.45 ± 1.03	0.57 ± 0.09	<0.0001	0.685
	80O_2_:20CO_2_	11.79 ± 1.37 ^b^	16.06 ± 4.00 ^a^	9.17 ± 0.85	0.50 ± 0.08	<0.0001	0.713
	70O_2_:30CO_2_	11.63 ± 1.21 ^b^	14.45 ± 4.39 ^a^	9.29 ± 1.04	0.40 ± 0.10	0.0010	0.505
	60O_2_:40CO_2_	11.50 ± 0.94 ^b^	14.88 ± 3.15 ^a^	9.65 ± 0.72	0.35 ± 0.07	<0.0001	0.629
	50O_2_:50CO_2_	11.70 ± 0.91 ^b^	15.58 ± 2.75 ^a^	9.76 ± 0.57	0.41 ± 0.05	<0.0001	0.785
	100O_2_	11.52 ± 0.88 ^b^	16.34 ± 4.81 ^a^	8.70 ± 1.05	0.55 ± 0.10	<0.0001	0.662
	VP	12.04 ± 1.65 ^b^	15.39 ± 4.11 ^a^	9.62 ± 0.98	0.43 ± 0.09	0.0000	0.582
Filtration time ^¥^	AA	33.89 ± 27.49 ^a^	NA	−3.54 ± 5.07	8.22 ± 0.93	<0.0001	0.918
	90O_2_:10CO_2_	17.33 ± 11.76 ^b^	42.32 ± 16.94 ^a^	3.90 ± 4.43	2.71 ± 0.39	<0.0001	0.747
	80O_2_:20CO_2_	17.78 ± 12.36 ^b^	35.22 ± 7.95 ^a,b^	8.87 ± 3.45	1.84 ± 0.31	<0.0001	0.693
	70O_2_:30CO_2_	18.67 ± 13.11 ^b^	31.03 ± 7.41 ^a,b^	14.02 ± 4.60	1.13 ± 0.41	0.0140	0.324
	60O_2_:40CO_2_	16.89 ± 11.67 ^b^	29.44 ± 9.08 ^a,b^	12.55 ± 4.59	1.11 ± 0.41	0.0150	0.316
	50O_2_:50CO_2_	17.22 ± 11.94 ^b^	29.17 ± 6.95 ^a,b^	12.97 ± 4.26	1.07 ± 0.38	0.0120	0.333
	100O_2_	18.56 ± 13.25 ^b^	38.07 ± 6.51 ^a^	9.89 ± 3.73	1.93 ± 0.33	<0.0001	0.678
	VP	17.33 ± 11.54 ^b^	22.54 ± 3.78 ^b^	12.55 ± 3.40	0.77 ± 0.30	0.0210	0.289

The results are expressed as the means ± standard deviation (*n* = 2). NA—Not applicable. ^a,b,c^ Different letters indicate significant differences (*p* < 0.05) between treatments. ^€^ AA (ambient air); 90O_2_:10CO_2_, 80O_2_:20CO_2_, 70O_2_:30CO_2_, 60O_2_:40CO_2_, 50O_2_:50CO_2_, and 100O_2_ (modified atmosphere packaging with 90%:10%, 80%:20%, 70%:30%, 60%:40%, 50%:50%, and 100%:0% of oxygen and dioxide carbon ratios, respectively); VP (vacuum packaging). ^¥^ TVB-N—total volatile basic nitrogen in mg for TVB-N/100g of meat; filtration time in min. ^£^ Values for the total amount of each physicochemical parameter during the storage period from day 0 to 9 (∆_0–9_) and from day 10 to 20 (∆_10–20_).

**Table 2:** Bacterial growth parameters of ground beef treated with different packaging systems stored at 2 °C for 20 days.

Treatments ^€^	Parameters ^£^	AMHB ^ψ^	APHB ^ψ^	Shelf Life * (Days)
AA	Lag	2.53 ± 0.02 ^g^	2.45 ± 0.00 ^e^	3
	GT	0.95 ± 0.01 ^e^	0.97 ± 0.00 ^g^	
	NC	8.67 ± 0.00 ^g^	8.84 ± 0.00 ^g^	
90O_2_:10CO_2_	Lag	4.04 ± 0.01 ^f^	1.98 ± 0.01 ^f^	6
	GT	2.35 ± 0.01 ^b^	1.82 ± 0.00 ^e^	
	NC	8.99 ± 0.00 ^d^	8.89 ± 0.00 ^f^	
80O_2_:20CO_2_	Lag	6.96 ± 0.03 ^c^	3.20 ± 0.00 ^c^	8
	GT	1.70 ± 0.01 ^d^	1.99 ± 0.00 ^d^	
	NC	8.94 ± 0.00 ^e^	8.95 ± 0.00 ^e^	
70O_2_:30CO_2_	Lag	4.97 ± 0.03 ^e^	4.20 ± 0.01 ^b^	7
	GT	2.76 ± 0.02 ^a^	1.72 ± 0.00 ^f^	
	NC	8.89 ± 0.00 ^f^	8.80 ± 0.00 ^h^	
60O_2_:40CO_2_	Lag	7.04 ± 0.03 ^b,c^	1.96 ± 0.00 ^f^	8
	GT	2.07 ± 0.02 ^c^	2.20 ± 0.00 ^c^	
	NC	9.29 ± 0.00 ^b^	9.37 ± 0.00 ^a^	
50O_2_:50CO_2_	Lag	7.99 ± 0.03 ^a^	4.26 ± 0.01 ^a^	8
	GT	1.59 ± 0.01 ^d^	1.82 ± 0.00 ^e^	
	NC	9.34 ± 0.01 ^a^	9.34 ± 0.00 ^b^	
100O_2_	Lag	6.63 ± 0.02 ^d^	1.96 ± 0.00 ^f^	7
	GT	2.18 ± 0.01 ^c^	2.37 ± 0.00 ^b^	
	NC	8.99 ± 0.00 ^c^	9.03 ± 0.00 ^d^	
VP	Lag	7.20 ± 0.14 ^b^	2.84 ± 0.00 ^d^	8
	GT	2.80 ± 0.08 ^a^	2.57 ± 0.00 ^a^	
	NC	8.39 ± 0.00 ^h^	9.03 ± 0.00 ^c^	

Results are expressed as the means ± standard deviation (*n* = 2). ^a^^–h^ Different letters in the same column indicate, within the same parameter, significant differences (*p* < 0.05) between treatments. ^€^ AA (ambient air); 90O_2_:10CO_2_, 80O_2_:20CO_2_, 70O_2_:30CO_2_, 60O_2_:40CO_2_, 50O_2_:50CO_2_, and 100O_2_ (modified atmosphere packaging with 90%:10%, 80%:20%, 70%:30%, 60%:40%, 50%:50%, and 100%:0% of oxygen and dioxide carbon ratios, respectively); VP (vacuum packaging). ^£^ Lag—lag phase (h); GT—generation time (h); NC—number of colonies in the stationary phase (Log cfu/g). ^ψ^ AMHB—aerobic mesophilic heterotrophic bacteria; APHB—aerobic psychrotrophic heterotrophic bacteria. * The time necessary for average AMHB count values to attain a threshold of 7.0 Log cfu/g [45] over the entire storage period of 20 days at 4 °C.
